# Clinical Management of Low Vitamin D: A Scoping Review of Physicians’ Practices

**DOI:** 10.3390/nu10040493

**Published:** 2018-04-16

**Authors:** Michelle Rockwell, Vivica Kraak, Matthew Hulver, John Epling

**Affiliations:** 1 Department of Human Nutrition, Foods, and Exercise, Virginia Polytechnic Institute & State University, Blacksburg, VA 24061, USA; vivica51@vt.edu (V.K.); hulvermw@vt.edu (M.H.); 2 Department of Family and Community Medicine, Virginia Tech Carilion School of Medicine and Research Institute, Roanoke, VA 24016, USA; jwepling@carilionclinic.org

**Keywords:** vitamin D, 25-hydroxyvitamin-D, 25-OH-D, low vitamin D, screening, physician practices, low value care, test overutilization

## Abstract

The role of vitamin D in the prevention and treatment of non-skeletal health issues has received significant media and research attention in recent years. Costs associated with clinical management of low vitamin D (LVD) have increased exponentially. However, no clear evidence supports vitamin D screening to improve health outcomes. Authoritative bodies and professional societies do not recommend population-wide vitamin D screening in community-dwelling adults who are asymptomatic or at low risk of LVD. To assess patterns of physicians’ management of LVD in this conflicting environment, we conducted a scoping review of three electronic databases and the gray literature. Thirty-eight records met inclusion criteria and were summarized in an evidence table. Thirteen studies published between 2006 and 2015 across seven countries showed a consistent increase in vitamin D lab tests and related costs. Many vitamin D testing patterns reflected screening rather than targeted testing for individuals at high risk of vitamin D deficiency or insufficiency. Interventions aimed at managing inappropriate clinical practices related to LVD were effective in the short term. Variability and controversy were pervasive in many aspects of vitamin D management, shining a light on physicians’ practices in the face of uncertainty. Future research is needed to inform better clinical guidelines and to assess implementation practices that encourage evidence-based management of LVD in adult populations.

## 1. Introduction

Vitamin D is an essential nutrient obtained by humans through sunlight exposure to ultraviolet B (UVB) light, dietary sources, and dietary supplements. Many factors influence the vitamin D status of individuals and populations including: latitude, season, time spent outdoors or in UVB light, clothing habitually worn, sunscreen use, weight status, skin color, and some medications and medical conditions [1]. People who are deficient in vitamin D may develop rickets, osteomalacia or other bone disorders.

Vitamin D is found naturally in only a few foods—fatty fish (i.e., salmon, tuna, and mackerel), egg yolks, certain mushrooms—and in dairy products, margarine, ready-to-eat cereals, and fruit juices that have been fortified. Supplemental vitamin D is available in a variety of over-the-counter (OTC) and prescription strengths, in both ergocalciferol (vitamin D_2_) and cholecalciferol (vitamin D_3_) forms, and for administration orally or via intramuscular injection. Vitamin D is fat soluble; therefore, a risk of toxicity may exist with excessive vitamin D treatment.

Blood levels of vitamin D are most commonly evaluated through measurement of serum 25-hydroxyvitamin-D (25-OH-D). While 1,25-dihydroxyvitmain D (1,25-OH-D) is the active form of vitamin D, it has a shorter half-life than 25-OH-D (hours vs. weeks); thus, 25-OH-D is considered the best clinical indicator of vitamin D status. Estimates of the incidence of population-wide vitamin D deficiency and insufficiency, referred to as low vitamin D (LVD) throughout this paper, vary widely. Holick [2] has described LVD as reaching pandemic proportions in populations, whereas other clinicians and researchers have asserted that LVD rates are overestimated or exaggerated [3,4]. Variability in estimates of LVD may be due to how it is defined, and blood level targets considered sufficient or optimal to support good health [1,5]. In 2011, an expert committee convened by the United States (U.S.) Institute of Medicine (IOM) (changed to the Health and Medicine Division of the National Academy of Medicine in 2016) reported that 25-OH-D of 20 ng/mL is sufficient to support bone health in 97.5% of the population [6]. In contrast, the U.S. Endocrine Society considers <20 ng/mL indicative of LVD [7]. Table 1 and Table 2 summarize the vitamin D screening and testing guidelines and recommendations from several authoritative bodies and professional societies in North America and Europe. Variations in clinical diagnosis of LVD in individuals/patients occur for various reasons, including conflicting professional recommendations and practice guidelines, unfamiliarity with recommendations and guidelines, independent clinical judgement, or the tendency to default to laboratory-testing target levels.

Daily requirements, treatment guidelines and protocols, and monitoring strategies for LVD are unclear, variable, contradictory, and sometimes poorly-defined. Additionally, many laboratory methods are used to quantify 25-OH-D (e.g., liquid chromatography-tandem mass spectrometry, enzyme linked immunosorbent assay, chemiluminescence immunoassay, and new point-of-care assays [15]) resulting in notable intra- and inter-assay variability. 

In recent years, the role of vitamin D in the prevention and treatment of numerous non-skeletal conditions and chronic diseases has gained attention. Cardiovascular disease, diabetes, some cancers, autoimmune disorders, infertility, and depression are among many conditions associated with LVD status [16,17,18]. More than 300 new PubMed entries for “vitamin D” or a similar term in the title have been made monthly since 2013. A majority of the research that links vitamin D status to non-skeletal issues or conditions is based on observational studies, theories, and newly discovered mechanisms rather than randomized controlled trials conducted in human populations. In 2011, the IOM revised the Dietary Reference Intakes (DRI) for vitamin D for populations (i.e., adequate intake for infants ages 12 months and younger (400 IU); estimated average requirement (400 IU) and recommended dietary allowance (600 IU) for children ages 1 year and older through adulthood). The U.S. Endocrine Society also published clinical guidelines for the Evaluation, Treatment, and Prevention of Vitamin D Deficiency that same year. However, only skeletal health research was used to inform these recommendations because the available research on non-skeletal conditions was considered insufficient or conflicting [6,7]. Debate exists regarding the role of vitamin D in non-skeletal conditions and the quality of data for some conditions has continued to evolve. Nevertheless, the U.S. Preventive Services Task Force (USPSTF), an independent panel of experts who issue evidence-based clinical practice recommendations, concluded in 2015 that there was insufficient evidence to support population-wide screening for individuals at low risk of vitamin D deficiency [8]. Improved health status has not been reported in asymptomatic individuals treated for LVD [19]. 

Emerging research and inconsistencies in clinical guidelines have captured the attention of the media, public, and healthcare providers [20]. Despite formal guidelines and recommendations suggesting otherwise, a significant increase in screening and testing for LVD has been reported [6,21,22]. Laboratory test overutilization and over diagnosis are recognized problems since both impact healthcare costs and quality of care [23,24]. A 2012 IOM report concluded that $750 billion annually (representing over 30% of total U.S. healthcare spending) is used for unneeded care, such as non-indicated laboratory testing. Efforts to curb this overutilization have included the Choosing Wisely campaign (www.choosingwisely.org) that outlines recommendations against vitamin D testing for low-risk patients [25,26]. 

The identification of existing and evolving clinical practice patterns associated with LVD in adult populations is necessary to design, implement, and evaluate interventions, such as Choosing Wisely, to reduce low value care. Numerous research studies and reports have assessed physicians’ practice patterns associated with LVD, but no overview or comprehensive summary of the clinical management of LVD and its implications has been published. This paper addresses this knowledge gap by reviewing the healthcare services literature regarding: (1) Physicians’ management of LVD in community-dwelling adults, (2) costs associated with physicians’ clinical practices related to LVD, and (3) efforts to constrain inappropriate clinical practices demonstrated by physicians related to LVD.

## 2. Materials and Methods

The research question that guided this review was: How are clinical practices regarding vitamin D impacted by the changing guidelines and research base concerning the management of LVD in community-dwelling adults? Due to the broad nature of the research question, a scoping review was selected to systematically assess and describe the published literature for clinical management, associated costs, and attempts to constrain physicians’ practices related to LVD in an unbiased and transparent manner, while identifying key themes and future research needs [27]. Vitamin D screening was defined as testing asymptomatic individuals for the presence of LVD, whereas vitamin D testing was defined as evaluating selected symptomatic or at-risk individuals for LVD. As a scoping review, this study sought to describe the breadth of the literature rather than to emphasize quality of the studies, and to determine the value and feasibility of undertaking a systematic review for a more focused research question related to this topic [28].

### 2.1. Search Strategy

The Cochrane Library scoping review methodology [28] and Preferred Reporting Items for Systematic Reviews and Meta-Analyses (PRISMA) checklist [29] informed the conduct of this scoping review. A literature search was performed by M.R. in consultation with a research librarian in November 2017. Three electronic databases (i.e., PubMed, EMBASE, and Cochrane) were searched between 1997 and 2017. The search start date was selected as 1997 when the previous U.S. recommended dietary allowance for vitamin D was established. The following MeSH search terms were used: “vitamin D” [title or abstract] AND (“physician” OR “healthcare provider” OR “manag*” OR “primary care” OR “general practice” OR “lab* test” OR “screen*” OR “prescri*” OR “cost” OR “economic” OR “attitude”) (all fields). An update search was conducted in January 2018 to identify any articles published since the original search. During this second search, the reference lists from included articles were scanned for additional relevant literature, and a gray literature search was conducted in January 2018 using Google and the search terms above.

### 2.2. Inclusion and Exclusion Criteria

Review involved scanning the title and abstract of each identified article for relevance to the research question. All articles written in the English language that related to vitamin D screening and testing in community-dwelling adults were included. Only articles focused on physicians were included because published articles related to vitamin D testing patterns for other health professionals and medical team members were limited (three were identified). However, in a few of the included articles, medical team members such as physicians’ assistants or nurse practitioners were grouped with physicians for analyses. Articles were excluded that focused exclusively on children, individuals living in residential care facilities, and those with specific medical conditions (e.g., osteoporosis, kidney disease, or multiple sclerosis). Cost evaluations were included if they assessed outcomes directly resulting from physicians’ management of LVD.

### 2.3. Data Extraction and Synthesis

Data were extracted and summarized in an evidence table that included population, setting, study methodology, and key findings. Articles were grouped by outcomes reported including: vitamin D laboratory testing patterns, costs associated with vitamin D testing, knowledge, attitudes and/or behaviors related to physicians’ management of vitamin D, and attempts to change physicians’ practices involving vitamin D. Some articles were grouped in more than one outcome. Specific quality assessments were not performed beyond noting the methodology in keeping with the purpose of this scoping review.

Throughout the study, vitamin D was reported as IU (1 IU = 0.025 µg) and blood 25-OH-D was reported as ng/mL (1 ng/mL = 2.5 nmol/L). When applicable, monetary data was reported in the currency used in the original source and converted to U.S. dollars using January 2018 exchange rates.

## 3. Results

**Figure 1:**
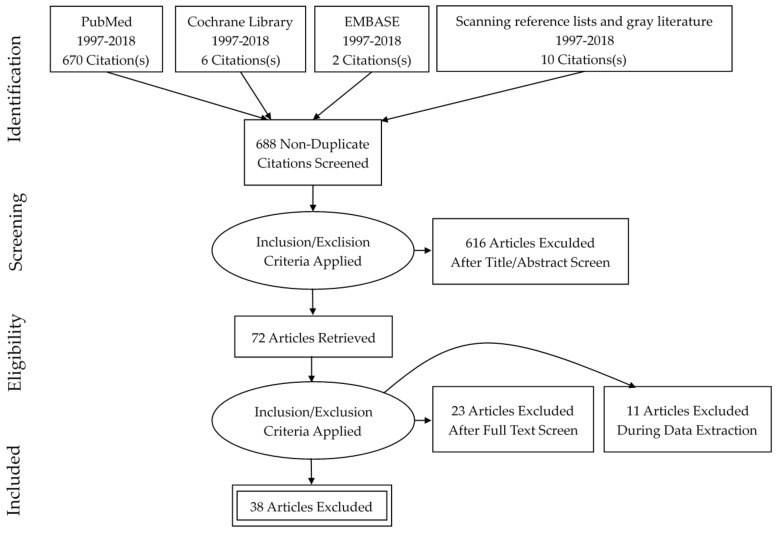
Preferred reporting items for systematic reviews and meta-analyses flow diagram for the scoping review.

Figure 1 shows the PRISMA flowchart for the scoping review. Of the 688 articles identified by the search, 72 met the initial inclusion criteria. An additional 34 articles were excluded after title and abstract review because clinicians, patients, the setting, or outcomes did not meet the inclusion criteria. The remaining 38 articles were included in this review [30,31,32,33,34,35,36,37,38,39,40,41,42,43,44,45,46,47,48,49,50,51,52,53,54,55,56,57,58,59,60,61,62,63,64,65,66,67,68]. Two gray literature documents were included within the final 38 articles.

### 3.1. Vitamin D Laboratory Testing

Trends in 25-OH-D laboratory tests are shown in Table 3. An increase in 25-OH-D testing was reported in six different countries: Australia, Canada, France, Saudi Arabia, United Kingdom, and the U.S. No articles reported that the rate of 25-OH-D testing decreased or stayed the same. A 94-fold increase in testing (over 4.5 million tests) was reported in Australia between 2006 and 2010 [30], 83-fold increase in tests in U.S. Medicare Part B recipients [31], 11-fold increase among primary care patients in Liverpool, United Kingdom [32], and nearly eight-fold increase (in 25-OH-D and/or 1,25-OH-D) in France based on nationally-representative health insurance data, totaling 18% of patient visits from 2008 to 2013 [33]. The volume of 25-OH-D tests increased by six-fold in a National Health Service hospital in London, United Kingdom and more than doubled in a large Scottish hospital from 2008 to 2010, creating a substantial laboratory backlog [34].

Initial tests represented most recorded tests [32,39,41,42]. One exception was reported by a U.S. Veterans Administration study in which over 70% of tests were repeat or follow-up tests [43]. Of studies evaluating repeat tests over time, a quarter of French patients incurred three or more tests in a five year period [32] while 27% of Australian patients incurred three or more tests in a four year period [30] and three or more 25-OH-D tests were ordered for patients in a hospital in Saudi Arabia within one year, with some patients incurring more than six tests [39]. Khalifa et al. [39] described three trends in their analysis of 25-OH-D testing patterns: (1) physicians ordered many initial tests in different patients; (2) physicians repeated tests in the same patient; and (3) some physicians demonstrated both 1 and 2. 

Minimal data regarding characteristics of physicians who order 25-OH-D tests are available. However, Tapley et al. [40] reported that Australian physician trainees were more likely to order tests if they worked within a practice that completely bulk bills the national insurance plan (no out-of-pocket or private insurance charges) or if they were ordering other laboratory blood tests. In 2006–2010, 80% of the 25-OH-D tests ordered throughout Australia were ordered by general practitioners and 20% were ordered by specialists [30]. Caillet et al. [33] reported an increase in proportion of 25-OH-D tests ordered by general practitioners in France from 2008 to 2013 (54% to 66%) and a concurrent decrease in 25-OH-D tests ordered by specialists (30% to 13%). 

Physicians were more likely to order 25-OH-D tests for female patients, older patients, and migrant patients [32,38,40,42,43,44,45,46]. Ages described as “older” varied by study with tested patients having a mean age of 50 years [32,46], 63 years [42], or older than 65 years [40]. Gowda et al. [38] reported that 25-OH-D testing increased with age throughout adulthood. Lower socioeconomic status was associated with higher likelihood of being tested in one study [33] but had no impact on test likelihood in another study [38]. Individuals classified as “visible minorities” were more likely to have 25-OH-D tests in one study [37]. 

Medical diagnoses associated with 25-OH-D testing were most commonly “health maintenance”, “medical check-up”, and “tiredness/lethargy/fatigue” in a 2010–2013 Australian cohort [40]. Bilinski and Boyages [30] evaluated how the 94-fold increase in 25-OH-D testing from 2006 to 2010 in Australia compared to more routine testing—e.g., complete blood count (CBC) orders. Orders for CBC increased only 2.5-fold, indicating that 25-OH-D testing increased at a significantly greater rate than orders for other tests. The number of bone densitometry tests ordered during the 2006-2010 timeframe increased just 2.5-fold. The same research team reported a 43.6-fold increase in 25-OH-D testing among 45–74-year-old females in Australia [35]. Because they noted only a concurrent 1.2-fold increase in bone densitometry testing, authors labeled this pattern “the Vitamin D Paradox”, as it appeared that 25-OH-D testing was not associated with evaluation of bone health [35]. Huang et al. [47] reported that 97.2% of the 7.5 million 25-OH-D tests ordered within a national U.S. outpatient cohort were coded as ICD-9 268.9, *unspecified vitamin D deficiency*, with less than three percent coded as *vitamin D deficiency-related osteomalacia* or *general vitamin D deficiency*.

The proportion of 25-OH-D tests results categorized as vitamin D deficient or insufficient ranged from 42% to 67% [32,41,42,43,45]. Of note, researchers used different cut-offs for *deficiency* and *insufficiency* and the *insufficiency* category was not always reported. For example, Zhao et al. [32] classified vitamin D deficiency as 25-OH-D <12 ng/mL and insufficiency as 12–20 ng/mL whereas Wei et al. [41] classified <20 ng/mL as deficiency and 20–30 ng/mL as insufficiency. Three studies did not include an insufficiency category in their analyses [42,43,45]. 

Five studies analyzed whether ordered 25-OH-D tests were medically indicated. It is difficult to compare the results of these studies because varying criteria and guidelines were used in analyses. Forty-eight percent of 25-OH-D tests ordered by physicians in an Australian health system during 2012 were not considered guideline-supported based on authors’ application of multiple professional guidelines [42]. Over 40% of 25-OH-D tests ordered for patients were covered by a private insurance company in upstate New York, U.S. but did not meet the company’s criteria for medically indicated [47]. Non-indicated tests comprised nearly 10% of 25-OH-D tests in a 2014 northeast U.S. analysis [44] and 8.2% of tests ordered by physicians in a research and teaching hospital in Italy from 2012–2014 [48], both based on respective national guidelines. In the later analysis, 1,25-OH-D was ordered for an additional 8% of patients, also deemed inappropriate by authors [48]. Only a fraction (3%) of 25-OH-D tests ordered in a California, U.S. managed care health system were classified as “high risk” (if patients were diagnosed with fat malabsorption, chronic kidney disease, HIV, anti-epileptic drug use, or had a history of bariatric surgery) [41].

### 3.2. Vitamin D Prescriptions

Assessing strategies for treating LVD is difficult because they may include either recommended dietary changes, increased UVB exposure, and/or vitamin D supplements obtained over-the-counter or by prescription. However, a 75-fold increase in vitamin D_3_ prescriptions was observed in Tuscany, Italy from 2006 to 2013 [49]. An eight-fold increase in vitamin D_2_ prescriptions was reported in California, U.S. Kaiser Permanente patients from 2007 to 2010 [50]. 

Prescribing patterns varied among physicians. For example, Caillet et al. [45] observed over 350 different treatment regimens administered to 1311 French patients in 2008 and 2009 while Pepper et al. [51] described 36 discrete vitamin D prescribing regimens within a Veterans Medical Center in Georgia, U.S. in 2003 to 2006. Vitamin D treatments varied by form (i.e., vitamin D_2_ vs. D_3_), mode of delivery (i.e., intramuscular injection vs. oral), dose and frequency, and length of treatment regimen. 

### 3.3. Physicians’ Knowledge, Attitudes, and Behaviors Related to Management of LVD

Physicians’ knowledge, attitudes, and behaviors related to vitamin D testing were evaluated by six studies. Three studies [52,53,54,55] administered adaptations of the same survey, “Prescribing Sunshine”, aimed at assessing the attitudes, practices, and knowledge regarding vitamin D and sun exposure among primary care physicians in Australia, New Zealand, and Saudi Arabia, respectively. Epling et al. [55] assessed primary care providers’ practice patterns involving vitamin D using focus groups, while Tarn et al. [56] analyzed recordings of patient-physician office visits, and Bennett et al. [57] explored physicians’ management of vitamin D through structured interviews. 

#### 3.3.1. Physicians’ Knowledge

Physicians’ confidence in their vitamin D knowledge varied, with 9–40% responding “not at all confident” in their vitamin D knowledge [52,53,54]. Information regarding vitamin D was obtained through multiple different sources and strategies. The study by Bennett et al. [57] reported prevalence of both passive and active information-seeking strategies, with few physicians reporting interactive strategies in obtaining vitamin D knowledge. Physicians in the Epling et al. [55] study discussed informal conversations with colleagues (not necessarily recent), point-of-care resources, professional guidelines, and scientific literature as information sources. Physicians in Saudi Arabia stated that continuous medical education, Internet resources, and medical journals were their primary information sources [54]. Australia released a national position statement regarding vitamin D and sun exposure in 2009, but only about 20% of physicians reported having read it when responding to a 2010 survey [53]. Bovisnki et al. [53] and Reeder et al. [52] both reported that about half of surveyed physicians agree with the statement “information about vitamin D is not readily available to general practice physicians”. Regardless, more than half of physicians in these two studies reported that the amount of information they were exposed to regarding vitamin D was “more than normal” in the previous year [52,53]. Very few physicians agreed that this information influenced their practice. Physicians in the Tarn et al. [56] study provided information to patients that was inconsistent with clinical guidelines regarding vitamin D screening in asymptomatic adults, the definition of LVD, and the optimal range for 25-OH-D. Nearly 100% of “Prescribing Sunshine” respondents strongly agreed that clear and concise guidelines regarding LVD would beuseful [52,53,54,55]. 

#### 3.3.2. Communication

The topic of vitamin D was raised in more than 15% of patient encounters in the study of Southern California, U.S. physicians [56]. Despite a great deal of uncertainty regarding vitamin D information and guidelines, physicians conveyed over 95% of vitamin D-related statements with certainty [56]. For example, some patients were told that vitamin D screening was routinely recommended despite insufficient evidence to support screening [56]. Bennett et al. [57] described physicians’ employment of Uncertainty Management Theory in conversations with patients about vitamin D treatment. 

#### 3.3.3. Testing and Treatment

Physicians varied in their beliefs and practices regarding testing for LVD, with some supporting screening for all their patients, others believing that testing should be based on risk factors (the definitions of these risk factors were highly variable), and others focusing minimally on testing [55,57]. Epling et al. [55] found that patient demand was a primary driver for vitamin D testing. However, only about 20% of “Prescribing Sunshine” respondents indicated that patients initiated testing [53].

The definition of deficient/adequate/optimal 25-OH-D levels and recommended treatment regimens varied broadly [55,56,57]. Treatment of LVD with dietary supplements was more commonly recommended than dietary changes or increased exposure to sunlight [52,53,54]. Confusion about the amount of sunlight exposure required for optimal vitamin D synthesis was expressed, in addition to concern about the association between excess sun exposure and skin cancer risk [52,53]. About 70% of responding physicians in Australia and New Zealand disagreed that “it is more important to stay out of the sun than get enough vitamin D” [52,53]. 

A variety of maladaptive responses to uncertainty surrounding vitamin D testing were reported. For instance, some physicians admitted manipulating diagnostic codes so vitamin D tests were more likely to be reimbursed by insurance [55]. Bennett et al. [57] discussed physicians’ tendency to craft certain statements and stories even when uncertainty exists. 

#### 3.3.4. Attitudes

Uncertainty, doubt, and skepticism regarding vitamin D management were themes in two studies [55,57]. Some physicians discussed their desire for patients to be proactive in their own care, yet also expressed frustration about the influence and unreliability of accessed media sources [57]. The issue of limited time for patient encounters was discussed, with some physicians mentioning that vitamin D management was not always the top priority in patient visits [55,57]. 

### 3.4. Economic Impact

The economic impact of vitamin D testing is sizable and increasing. Table 4 includes studies and reports which have analyzed or estimated direct costs of vitamin D testing. For example, Bilinski and Boyages [58] reported that nearly $100 million (Aus.)/$794 million (U.S.) was spent on vitamin D testing in Australia in 2010, a value that reflects 1% of national healthcare spending. In the U.S., $224 million was spent on vitamin D testing for Medicare patients (individuals over 65 years of age or qualifying based on disability) and $33 million was spent on 2014 vitamin D tests among privately insured patients in Upstate New York, U.S. [47]. Over $20 million of “unnecessary” testing was identified in Virginia, U.S. in 2014 based on analysis using health waste calculator software [59]. The $20 million represents approximately 0.9% of the state’s healthcare spending in 2014, up from 0.4% in 2013 [60]. Non-indicated vitamin D tests were more common in U. S. Medicare patients than commercially insured patients based on Medicare guidelines for vitamin D testing (13% vs. 8% of patients seen from 2009-2011, respectively) [35]. No studies reported a decrease in vitamin D testing. 

Patients diagnosed with LVD in U.S. Veteran’s Medical Centers used more healthcare services and incurred higher medical costs than patients not diagnosed [43,61]. Vitamin D status also correlated with increased hospitalization and medical costs in generally healthy German adults [62]. Decreased muscle relaxant and pain medication prescriptions were associated with vitamin D status and supplementation in French patients dealing with chronic pain [47]. 

### 3.5. Efforts to Constrain Inappropriate Clinical Practice Related to Low Vitamin D

Interventions aimed at reducing inappropriate vitamin D test-ordering have been impactful. For example, the national health systems in France and Ontario, Canada restricted testing to only a subset of high-risk conditions [63,64]. Through reimbursing 25-OH-D testing only for osteoporosis/osteopenia, rickets, malabsorption syndromes, renal disease, and concurrent medications which may affect vitamin D metabolism, officials in Ontario predict a savings of approximately $65 million annually [64]. Deschasaux et al. [65] recommended a screening questionnaire, the vitamin D insufficiency prediction score, as an effective tool for identifying patients at high-risk for LVD and as a precursor for 25-OH-D testing while a Utah, U.S.-based team suggested benchmarking as an effective method of monitoring vitamin D testing [66]. Implementation of three clinical decision support tools in the electronic medical record of a large U.S.-based health system resulted in a 13% reduction in tests considered unnecessary by the health system’s evidence-based guidelines [67]. White et al. [68] also showed a decrease in inappropriate test-ordering through electronic medical record modification in two U.S. medical facilities. Direct physician feedback reduced inappropriate repeat 25-OH-D testing by 25% in Italy [69]. For example, physicians received a phone call and computer message when ordering a repeat 25-OH-D test less than 90 days after the previous 25-OH-D test [69]. Finally, patient and clinician education were shown to be effective in reducing the ordering of 25-OH-D tests [65,70].

## 4. Discussion

This scoping review identified literature related to physicians’ clinical management of LVD, costs associated with physicians’ clinical management of LVD, and efforts to constrain inappropriate clinical management of LVD by physicians in a variety of developed countries. Vitamin D laboratory testing, prescriptions, and costs associated with these practices have increased, in some cases dramatically, over the past 10–15 years. Patterns of test overutilization were demonstrated throughout reviewed studies. Interventions designed to constrain inappropriate clinical management patterns have produced promising results. 

Although a substantial volume of patients with LVD were identified through 25-OH-D testing, the odds of detecting LVD decreased. Reported increases in vitamin D testing were disproportionate to increases in other laboratory tests. Most articles reported testing patterns indicative of vitamin D screening. These patterns are inconsistent with the clinical guidelines and recommendations from USPSTF, IOM, U.S. Endocrine Society, and others (Table 1 and Table 2) who recommend vitamin D testing only for symptomatic patients or those at high risk of LVD. Billinski and Boyages [35] showed that vitamin D testing was not associated with bone-related diagnoses, which are commonly considered indicative of vitamin D testing. It is unknown, however, what proportion of tests were associated with other problems or diagnoses which may be considered high risk for LVD, such as chronic renal disease or malabsorption. Ambiguity and inconsistencies in LVD treatment guidelines may explain the excessive number of repeat vitamin D tests ordered in a short timeframe in some analyses. 

As noted in Table 4, the cost of rising 25-OH-D testing is significant. It could be argued that spending on 25-OH-D testing is trivial since it contributes marginally to total healthcare spending. However, achieving the global goal to contain healthcare spending, in part, by reducing low value care and medical waste will require collective effort at all levels of care and all levels of spending. Better management of vitamin D may serve as an example for future efforts to achieve higher value care. 

Costs reported in Table 4 do not include downstream costs associated with increased testing such as increased laboratory personnel, time/personnel needed to communicate test results to patients, tests ordered as follow-ups to initial testing, and treatment expenses. Minimal information is available about resource utilization related to increased vitamin D prescriptions and the variation in treatment patterns identified by this review.

Although increased healthcare costs were associated with LVD, it is difficult to determine if patients in these studies incurred higher healthcare costs only due to LVD. Since numerous factors are related to both LVD and poor health, patients with LVD may have been sicker than those without LVD. Rather than LVD causing health problems (and thus, higher costs), it is feasible that other health problems resulted in LVD.

Authors of several reviewed articles concluded that the standardization of guidelines and procedures regarding vitamin D testing and medical management would be valuable. Almost all “Prescribing Sunshine” respondents agreed that clear and concise guidelines were needed, with over 50% indicating their perception that information about vitamin D is not readily available to general physicians [52,53]. However, guidelines and recommendations from multiple expert bodies and professional associations exist (Table 1 and Table 2). Data collection for some studies occurred before Table 1 and Table 2 guideline and recommendations were published, so it is possible that physicians may have changed their vitamin D management after reviewing revised professional guidelines. Inconsistency in published guidelines and recommendations coupled with the recent intense focus on the role of vitamin D in non-skeletal conditions may explain the wide variation in management of LVD. Physicians’ lack of awareness of existing guidelines may also contribute to inconsistencies.

Aa better understanding is needed of the proportion of physicians who have reviewed the guidelines and recommendations included in Table 1 and Table 2. Finally, perhaps some physicians were aware of guidelines but did not agree with them, preferred to make decisions based on their own clinical judgement, or were influenced by the high volume of reports related to non-skeletal effects of LVD [71,72,73]. 

Epling et al. [55] discussed physicians’ practice patterns regarding vitamin D as set within clinical “mindlines”. Mindlines have been defined as ‘collectively reinforced, internalized tacit guidelines” [74] that arise from the interaction of knowledge, practice patterns and constraints, and the larger context of patient demand and the medical community. These mindlines may explain the noted contradictions in guidelines and physician practices. We found differences in the impact of patient demand on vitamin D test ordering [53,55]. Overall, a better understanding of the factors that influence the clinical management of LVD is needed. 

The issue of uncertainty was repeatedly cited as a highly influential contributor to excessive low value care, including 25-OH-D testing in low risk patients. Colla et al. [75] reported that over 60% of surveyed physicians found uncertainty involved in providing care disconcerting. Bennet et al. [57] described several communication and coping strategies employed by physicians in relation to uncertainty in vitamin D management. Other influences potentially include: defensive behavior/fear of malpractice accusations, responding to patients’ or family members’ demands, ease of ordering and obtaining test results, profit for medical subspecialties, clinical performance measures, and lack of feedback regarding cost and prevalence of testing. The allure of identifying an easy-fix or “magic pill” for patient treatment (i.e., treating LVD, recommending vitamin D supplementation) may be appealing to patients and physicians alike, contributing to vitamin D lab test overutilization. 

Some physicians noted conflict regarding multiple health goals and initiatives. For instance, the challenge of promoting UVB exposure to improve vitamin D status while recommending limited UVB exposure as a skin cancer precaution. Guidelines and tools for recommending appropriate sun exposure for different individuals in a variety of regions would be valuable to clinicians. Finally, with the average primary care visit lasting an average of 13–16 min [76], time to adequately address topics such as vitamin D may be limited, particularly in complex patients. One physician expressed practical challenges in translating medical recommendations in clinical practice given multiple constraints, stating “In training, the most important lesson they teach you is when not to do something. But in real life, it’s all about staying out of trouble, surviving, and keeping it quick” [73].

Multiple interventions led to meaningful reductions in inappropriate 25-OH-D test-ordering in the short term. However, evidence of long-term effectiveness is needed, in addition to physicians’ acceptance of these interventions, is needed. 

### 4.1. Future Research

High-quality evidence regarding whether or not vitamin D testing and/or treatment in asymptomatic adults improves health status or the economic bottom line is the priority for further research related to clinical management of vitamin D. Once this information is elucidated, methods for constraining test variation, improving adherence to guidelines, and reducing the cost of testing would appropriately be considered. Understanding more about why physicians provide increasing amounts of low value care—especially low cost, low value care—and how they experience uncertainty and emerging information may provide perspective into effective intervention for vitamin D management in addition to other health services. 

### 4.2 Study Strengths and Limitations

This study is the first review of literature related to clinical management of LVD. As is appropriate for the intent of a scoping review, the included evidence is heterogeneous in clinical setting, research methods, and analysis. Limitations of this review include the restriction to English language articles, and the lack of detailed critical appraisal of the included studies. Literature included in the review includes studies which took place at different points in time relative to published guidelines. Additionally, the researchers may have had different baseline assumptions for what constitutes appropriate management of LVD.

## 5. Conclusions

Evidence regarding the role of vitamin D in the prevention and treatment of non-skeletal conditions continues to evolve. The impact of vitamin D screening for asymptomatic or low-risk patients is unknown. Nevertheless, physician practice, as demonstrated in a variety of studies, is widely inconsistent, and includes many examples of non-indicated testing and overutilization. Clinical practice has surpassed available supporting evidence. Broad variability in physicians’ knowledge, attitudes, and behaviors related to vitamin D testing are reflective of the landscape of uncertainty in research findings, recommendations, and guidelines. Future research is needed to inform better clinical guidelines in this area, and to assess implementation practices that will encourage evidence-based management practices for LVD in adult populations. Moreover, greater understanding of physician management of uncertainty in clinical practice may help avoid overutilization and inconsistent practice in similar clinical situations.

## Figures and Tables

**Table 1:** Vitamin D screening and testing guidelines and recommendations by authoritative bodies and professional societies.

Recommendation	Population-Wide 25-OH-D Screening Recommended?	25-OH-D Testing for Individuals at High Risk of Deficiency Recommended?	Definition of “High Risk”
American Academy of Family Physicians [8]	Current evidence is insufficient to assess the balance of benefits and harms of screening for vitamin D deficiency (I)	No	N/A
Canadian Medical Association [9]	No	Yes	Significant renal or liver disease Osteomalacia, osteopenia or osteoporosis Malabsorption syndromes Hypo or hypercalcemia/ hyperphosphatemia Hypo or hyperparathyroidism Patients on medications that affect vitamin D metabolism or absorption Unexplained increased levels of serum alkaline phosphatase Patients taking high doses of vitamin D (>2000 IU daily) for extended periods of time (>6 months), and who are exhibiting symptoms suggestive of vitamin D toxicosis (hypervitaminosis D)
Central European Scientific Committee on Vitamin D [10]	No	Yes	Rickets, osteomalacia, osteoporosis, musculoskeletal pain, history of fracture or falls Calcium/phosphate metabolism abnormalities Hyperparathyroidism Malabsorption syndromes At-risk medications Dietary restriction, parenteral nutrition, eating disorder Kidney disease (stages 3–5) or transplant, liver disease, autoimmune disease, cardiovascular disease, some cancers, some infections
Kidney Disease Outcomes Quality Initiative (KDOQI) [11]	No	Yes	Stage 3–5 kidney disease, particularly if on dialysis
U.S. Endocrine Society [7]	No	Yes	Rickets, osteomalacia, osteoporosis Chronic kidney disease Hepatic failure Malabsorption syndromes Certain medications African-American and Hispanic children and adults Pregnant and lactating women Older adults with history of falls or non-traumatic fractures Obese children and adults Granuloma-forming disorders Some lymphomas
U.S. Preventive Services Task Force [12]	Current evidence is insufficient to assess the balance of benefits and harms of screening in asymptomatic adults (I statement)	N/A	N/A

* KDOQI changed diagnostic criteria for stage 3 kidney disease in 2003 resulting in more stage 3 kidney disease diagnoses and subsequent 25-OH-D tests. N/A = not available or not applicable.

**Table 2:** Blood 25-hydroxyvitmamin D (25-OH-D) levels indicative of vitamin D deficiency, insufficiency, adequacy, and toxicity.

Recommendation	Vitamin D Deficiency (25-OH-D)	Vitamin D Insufficiency (25-OH-D)	Adequate Vitamin D (25-OH-D)	Toxicity (25-OH-D)
Australian and New Zealand Bone Mineral Society/Endocrine Society of Australia and Osteoporosis Australia [13]	Mild deficiency: 12–19.5 ng/mL Moderate deficiency: 5–12 ng/mL Severe deficiency: <5 ng/mL		20 ng/mL at the end of winter; 24–28 ng/mL at the end of summer to allow for seasonal decrease	Not defined
Central European Scientific Committee on Vitamin D [10]	<20 ng/mL	20–30 ng/mL	30–50 ng/mL	>100 ng/mL
National Academy of Medicine (formerly IOM) [6]	<12.5 ng/mL	Not defined	12–20 ng/mL 25-OH-D of 20 ng/mL is sufficient to meet needs of 97.5% of the population	>50 ng/mL
Public Health England/National Osteoporosis Society [14]	<10 ng/mL	10–19.5 ng/mL	>20 ng/mL	Not defined
U.S. Endocrine Society [7]	<20 ng/mL	20–30 ng/mL	>30 ng/mL	>150 ng/mL

**Table 3:** Studies reporting trends in vitamin D testing patterns.

Study	Population	Setting	Time Frame	Key Findings
Bilinski and Boyages, 2013 [30]	2.4 million patients who received 25-OH-D tests (national health system data)	Australia	4-year period 2006–2010	94-fold increase in tests
Bilinski and Boyages, 2018 [35]	Women, ages 45–74 (national health system data)	Australia	10-year period 2001–2011	44% increase in tests
Caillet et al., 2017 [33]	639,163 patients (national health insurance database)	France	1-year period 2008–2009	18.5% were tested
Colla et al., 2017 [36]	Medicare and commercially insured patients (Health Care Cost Institute database)	United States	2-year period 2009–2011	10–16% of Medicare patents and 5–10% of commercially insured were tested
de Koning et al., 2014 [37]	Adult residents of 1436 census regions	Alberta, Canada	1-year period 2010–2011	8% were tested
Gowda et al., 2016 [38]	2187 patients seen in community health center	Melbourne, Australia	2-year period 2010–2012	56% of patients were tested
Khalifa et al., 2016 [39]	Hospital patients (King Faisal Hospital and Research Center)	Jeddah, Saudi Arabia	1-year period 2014–2015	30% increase in tests
Tapley et al., 2015 [40]	General practice patients (Recent cohort study)	4 states in Australia	3-year period 2010–2013	1% of patients were tested
Wei et al., 2014 [41]	22,784 managed care patients	California, United States	2-year period 2011–2013	11% of patients were tested
Zhao et al., 2015 [32]	Primary care patients	Liverpool, United Kingdom	5-year period 2007–2012	11-fold increase in tests

**Table 4:** Cost of vitamin D testing.

Study/Report	Population	Setting	Timeframe	Key Findings
Bartells, 2014 [47]	Commercially insured adult patients	Upstate New York, U.S.	1-year period 2014	$33 million spent on 25-OH-D tests
Bilinski and Boyages, 2013 [30]	Adults (national health system data)	Australia	4-year period 2006–2010	$20 million (Aus.)/$16 million (U.S.) spent on “non-indicated” 25-OH-D tests
Bilinski and Boyages, 2013 [35]	Women, ages 45–74 (national health system data)	Australia	10-year period 2001–2011	$7 million (Aus.)/$555,492 (U.S.) spent on 25-OH-D tests in 2001 and $40.5 million (Aus.)/$32 million (U.S.) in 2011
Caillet et al., 2016 [33]	All individuals (national health insurance database)	France	2-year period 2009–2011	€27 million/$33 million (U.S.) in 2009 to €65 million/$79 million (U.S.) on 25-OH-D tests
Cianferotti et al., 2015 [49]	Adults (20–90)	Tuscany, Italy	7-year period 2006–2013	€3.2 million/$3.9 million (U.S.) in 2006 to €8.2 million/$10.1 million (U.S.) in 2013 on 25-OH-D tests
Colla et al. 2015 [26]	Medicare patients (>65 years of age, qualify based on disability)	U.S.	5-year period 2006–2011	$224 million in 2011, average of $198 million/year 2006–2001 on 25-OH-D tests
Fairfield, 2017 [44]	All individuals without high risk diagnosis (ex: osteoporosis, malabsorption, liver disease, etc.)	Maine, U.S.	2-year period 2012–2014	$9,596,000 spent on “non-indicated” on 25-OH-D tests
